# Hammering Does Not Fit Fitts' Law

**DOI:** 10.3389/fncom.2017.00045

**Published:** 2017-05-29

**Authors:** Tadej Petrič, Cole S. Simpson, Aleš Ude, Auke J. Ijspeert

**Affiliations:** ^1^Biorobotics Laboratory, École Polytechnique Fédérale de LausanneLausanne, Switzerland; ^2^Department of Automatics, Biocybernetics and Robotics, Jožef Stean InstituteLjubljana, Slovenia; ^3^George W. Woodruff School of Mechanical Engineering, Georgia Institute of TechnologyAtlanta, GA, United States; ^4^Mechanical Engineering Department, Stanford UniversityStanford, CA, United States

**Keywords:** motor control, biomechanics, upper extremity, optimal control, arm movement, impact, Fitts' Law

## Abstract

While movement is essential to human wellbeing, we are still unable to reproduce the deftness and robustness of human movement in automatons or completely restore function to individuals with many types of motor impairment. To better understand how the human nervous system plans and controls movements, neuromechanists employ simple tasks such as upper extremity reaches and isometric force tasks. However, these simple tasks rarely consider impacts and may not capture aspects of motor control that arise from real-world complexity. Here we compared existing models of motor control with the results of a periodic targeted impact task extended from Bernstein's seminal work: hammering a nail into wood. We recorded impact forces and kinematics from 10 subjects hammering at different frequencies and with hammers with different physical properties (mass and face area). We found few statistical differences in most measures between different types of hammer, demonstrating human robustness to minor changes in dynamics. Because human motor control is thought to obey optimality principles, we also developed a feedforward optimal simulation with a neuromechanically inspired cost function that reproduces the experimental data. However, Fitts' Law, which relates movement time to distance traveled and target size, did not match our experimental data. We therefore propose a new model in which the distance moved is a logarithmic function of the time to move that yields better results (*R*^2^ ≥ 0.99 compared to *R*^2^ ≥ 0.88). These results support the argument that humans control movement in an optimal way, but suggest that Fitts' Law may not generalize to periodic impact tasks.

## Introduction

Movement is essential to human wellbeing. However, the control of movement is a very difficult problem. To produce deft and robust movements, the human nervous system must continuously control over 600 muscles while handling nonlinearities, nonstationarities, delays, noise, and uncertainties (Franklin and Wolpert, 2011). Despite these difficulties, humans move with apparent ease. However, human motor capability may become impaired due to age, illness, or injury. Robotic systems are also faced with many of the same challenges (Egeland et al., 1991; Park, 2002; Guigon et al., 2007; Peters et al., 2009), but meet with much less success than their healthy human counterparts (Yang et al., 2011; Vanderborght et al., 2013). A better understanding of the roles that the nervous and musculoskeletal systems play in producing movement will likely lead to advances in rehabilitation and robotic control.

Many neuromechanists employ simple tasks to study the nervous system in action under controlled conditions. Isometric tasks in which subjects interact with an immoble force sensor and reaching tasks in which the hand is moved from one point to another are commonly used to study sensorimotor learning (Rotella et al., 2015), movement control (Fitts, 1954), and neurophysiology (Shadmehr and Krakauer, 2008) in the upper extremity. Subjects may also be asked to interact with robotic co-workers that can record reaching dynamics (Burdet et al., 2001), generate disturbances, or create force fields (Shadmehr and Mussa-Ivaldi, 1994) during these tasks. When carefully considered, these experiments can provide a wealth of information on how the nervous system controls movement. However, these tasks are greatly simplified from real-world tasks. To study more complex tasks, some researchers have developed simple games, such as conkers, to study sensoriomotor learning (Sternad et al., 2011). However, even these studies simplify real-world tasks and rarely consider certain features of real-world tasks such as impacts.

Despite many possible ways to perform most tasks (Bernstein, 1967), upper extremity movements are highly stereotyped. Researchers note consistent characteristics such as bell-shaped velocity curves (Hollerbach and Atkeson, 1987; Berardelli et al., 1996) and speed-accuracy tradeoffs characterized by Fitts' Law (Fitts, 1954; Bootsma et al., 2004; Zhai et al., 2004). Fitts' Law expresses the time to complete a reach as a logarithmic function of the size of the target and the distance to the target (see Equation 1). In experiments relating to Fitts' Law, the kinematics (the beginning and final position of the arm or cursor) are prescribed and the subject is left to determine the time to reach. In certain periodic movements however, the time to complete an upper extremity movement can be specified and the subject left to determine the kinematics.

Movement is constantly refined by biological processes such as learning and evolution (Todorov, 2004). Because of this constant refinement, many researchers note that optimal control models utilizing cost functions such as minimum variance (Harris and Wolpert, 1998), minimum effort (Crowninshield and Brand, 1981), minimum jerk (Flash and Hogan, 1985), and minimum torque change (Uno et al., 1989) can be excellent models for the nervous system. In fact, many of the observed stereotypical behaviors discussed in the previous paragraph can be explained by optimality principles. Optimal control models have been used to reproduce human-like behaviors such as reaches (Todorov and Li, 2005), walking (Anderson and Pandy, 2001), and jumps (Anderson and Pandy, 1999; Ong et al., 2016). Though occasionally studied (Côté et al., 2008; Müller and Sternad, 2009), one activity that remains conspicuously unmodeled is Bernstein's hammering task (Bernstein, 1967; Müller and Sternad, 2009) that inspired much research into motor control and learning.

Here we extend Bernstein's hammering task into a targeted periodic impact task. We recorded impact forces and upper extremity kinematics in hammering. In order to examine how hammering strategies might change with different conditions, we used a set of hammers with different physical properties (hammer face area and mass) and prescribe different hammering frequencies. We hypothesized that hammering impact velocity and maximal height attained are the result of a tradeoff between maximizing task performance (quantified here as a maximal impact velocity) and minimizing effort (Crowninshield and Brand, 1981; Nelson, 1983). In order to test whether the mechanics of this task adhere to current theories in optimal human motor control, we implemented a feedforward optimal controller (Todorov, 2004) on a planar torque-driven 3-segment dynamical model of the upper extremity holding a hammer (**Figure 8**) using model parameters from Winter (2009). Our results show that humans appear to select optimal impact velocities that reflect a tradeoff between accomplishing the task and minimizing effort that do not adhere to Fitts' Law.

## Methods

### Subjects

Ten healthy male volunteers (age = 27.6 ± 3.6 years, height = 176.9 ± 5 cm, weight = 77.7 ± 11.2 kg) participated in the study. All subjects were right-handed and had no known neuromotor or sensory disorders (self-reported). Prior to their participation, subjects were informed of the course of study and gave their written informed consent in accordance with the code for ethical conduct in research at the Swiss Federal Institute of Technology (EPFL). This study was approved by the EPFL Human Research Ethics Committee (HREC No.: 008-2015/17.08.2015).

### Experimental protocol

**Figure 1:**
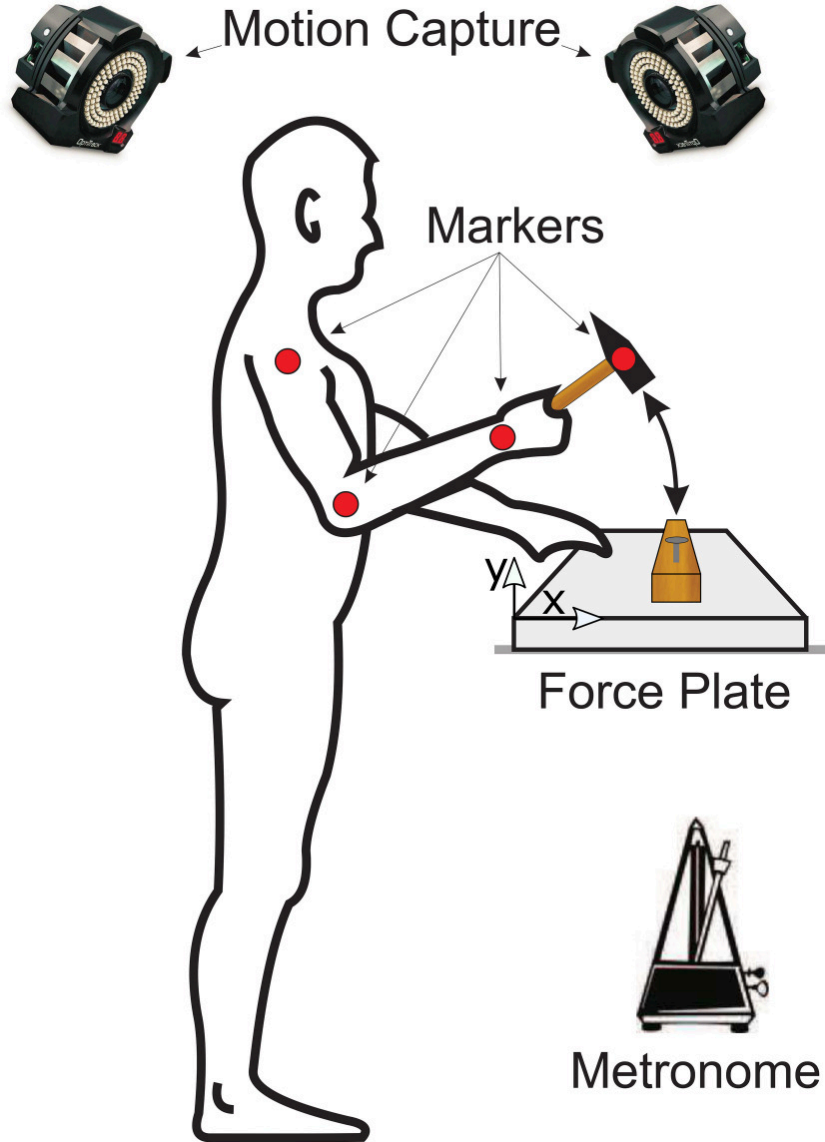
Experimental setup of the study. Each subject was asked to stand in front of a table on top of which a wooden board was placed on a force plate. Subjects were given one of four differently sized and weighted hammers at random and asked to drive a nail into the wooden board. The hammering frequency was controlled by asking each subject to match their hammer strikes with the clicks of a metronome. The forces on the wooden board were recorded by the force plate and the kinematic motion of the subjects' arms and of the hammer were recorded using an optical motion capture system.

Each subject was asked to step in front of a table on top of which was a wooden board mounted on a force plate (Kistler Instrument AG, Winterhur, Switzerland) as shown in Figure 1. Subjects were given one of four differently sized and weighted hammers (Table 1) and asked to drive a pre-started nail, i.e., a nail that had previously been driven to the point at which it would stand on its own, into the wooden board while matching their hammer strikes to the clicks of a metronome. Please note that subjects were not explicitly instructed to strike with their maximum impact speed but were allowed to self-select the best impact speed for their skill level. The metronome was set to one of five frequencies: 1, 2, 3, 4, or 5 Hz. The hammer used and metronome frequency for each trial were randomized. Note that subjects were not allowed to do a training trial first, but we assume that the random trial order cancels any learning effects. Subjects were allowed to use their nondominant hands to stabilize the wooden board. In each trial, the forces on the wooden board and the kinematic motion (14 Prime Series cameras, OptiTrac, USA) of the upper extremity and hammer were recorded at 1 kHz and 250 Hz, respectively. After completing the experimental trials, subjects were asked to subjectively rank each of the hammers in order from most to least preferred.

**Table 1:** Specifications of the hammers used in these experiments.

**Hammer**	**Face size [cm × cm]**	**Weight [kg]**
Small heavy	1.4 × 1.4	0.402
Small light	1.4 × 1.4	0.218
Big heavy	2.2 × 2.2	0.394
Big light	2.2 × 2.2	0.217

### Data processing

Statistical analyses were performed using the Statistics and Machine Learning Toolbox in Matlab. We calculated the hammer velocity, average maximal heights of the hammer, average times required for the hammer to go from maximal height to impact, and average maximal impact forces during hammering for each subject. We then used these average values from each subject for statistical analyses. We investigated the effects of time to impact, maximum height of the movement, and maximal force normalized with the hammer weight using two-way repeated-measures ANOVA with independent variables [hammers(4)×(frequency(5)]. The effect of maximum height of the movement, and maximal force normalized with the hammer weight for each combination of hammers and frequency was further determined using one-way repeated measures ANOVA. The differences between maximal heights and the differences between the normalized maximum forces at impact were tested with *post-hoc t*-tests with Bonferroni correction. The level of statistical significance used was 0.05 for all statistical tests.

### Modeling

In order to determine whether human hammering strategies adhere to Fitts' Law (Fitts, 1954; Bootsma et al., 2004; Zhai et al., 2004), we attempted to fit Fitts' model,

(1) $$\begin{array}{r} T_{f}=a+b\cdot log_{2}\left(2D/W\right), \end{array}$$

with data collected in our experiment. In this formulation, the movement time, *T*_*f*_, is a function of the distance from the hammer at peak height to the nail, *D*, and the face width of the hammer, *W*. The values of *a* and *b* were selected using a least squares difference regression.

In order to examine whether the human nervous system uses optimality principles to control hammering movements, we employed a feedforward optimal controller on two joints (shoulder and elbow) while the wrist was maintained at a desired position with an impedance controller. The human arm holding a hammer was modeled as a 3 link torque-driven robot operating in the saggital plane (**Figure 8**, right-hand column) whose parameters were computed based on data from Winter (2009) (see Appendix for more details) and whose dynamics are given by

(2) $$\begin{array}{r} \tau +JTF_{e}=H\left(q\right)\ddot{q}+h\left(q,\dot{q}\right)+g, \end{array}$$

where τ is a vector of joint torques, *q*, $\dot{q}$, and $\ddot{q}$ are vectors describing the joint angular position, velocity, and acceleration respectively, ***H***(*q*) is the inertia matrix, $h\left(q,\dot{q}\right)$ consists of the Coriolis, centrifugal, and viscous friction force vectors, *g* is the gravity force vector, *F*_*e*_ is a vector representing external forces (zero throughout the simulation), and ***J**^T^* is the transpose of the Jacobian matrix. The model was simulated in Matlab using a time step of 0.001 s beginning at the instant after one impact and terminating at the time of the next impact.

Human hammering is a difficult control task due to the need to balance energy transfer to the nail with accuracy. We hypothesize that the human nervous system determines an optimal tradeoff between maximal impact velocity (complete the task in the most effective manner) and minimal effort (Crowninshield and Brand, 1981; Nelson, 1983; Missenard and Fernandez, 2011). We thus determine the optimal joint torques by minimizing the cost function,

(3) $$\begin{array}{r} Cost=\left(1-\alpha \right)\frac{\sum_{i=1}^{n}\sum_{j=1}^{T}\tau_{i,j}^{2}}{C_{\tau_{max}}}-\alpha \frac{y_{T-1}-y_{T}}{C_{{ẏ}_{max}}}, \end{array}$$

where τ_*i,j*_ represents joint torques for *i* = 1, …, *n* joints over *j* = 1, …, *T* discretized time points, *y*_*T*_ and *y*_*T*−1_ are the vertical positions of the hammer head at the last and second-to-last time points of the simulation, 0 ≤ α ≤ 1 was designed as an expertise factor to represent the tradeoff in relative emphasis between impact velocity and effort (large α places more emphasis on energy transfer to the nail and a small α places more emphasis on effort conservation), *C*_τ_*max*__ is a scaling factor representing maximal effort (i.e., if maximal torque is applied for the duration of the simulation), and *C*_ẏ_*max*__ is a scaling factor representing the maximum achievable impact velocity. We compute *C*_τ_*max*__ as the discrete integral of the joint torque limits (whichever direction has the larger magnitude) over the length of the simulation and *C*_ẏ_*max*__ by simulating a hammer trajectory in which α = 1 and *C*_ẏ_*max*__ = 1. Because maximum effort and final velocity depend on the length of the simulation, we computed unique values of *C*_ẏ_*max*__ and *C*_τ_*max*__ for each hammering frequency. We constrain the model so that the hammer hits the same place in subsequent impacts ((*x*_0_, *y*_0_) = (*x*_*T*_, *y*_*T*_)) and there is no initial velocity ((ẋ_0_, ẏ_0_) = (0, 0)). We match the initial posture (location of (*x*_0_, *y*_0_) relative to the simulated shoulder) to the average posture used by our subjects determined by inverse kinematics. The terms, *C*_τ_*max*__ and *C*_ẏ_*max*__, are scaling factors included to facilitate direct comparison of the two terms making up the cost function, minimum effort and maximum final impact velocity. In order to determine whether the parameter, α, is constant within or across individuals, contours of constant α were generated and compared with experimental results. The optimal joint torques were determined using the interior point method implemented with the Matlab Optimization Toolbox.

## Results

### Experimental results

**Figure 2:**
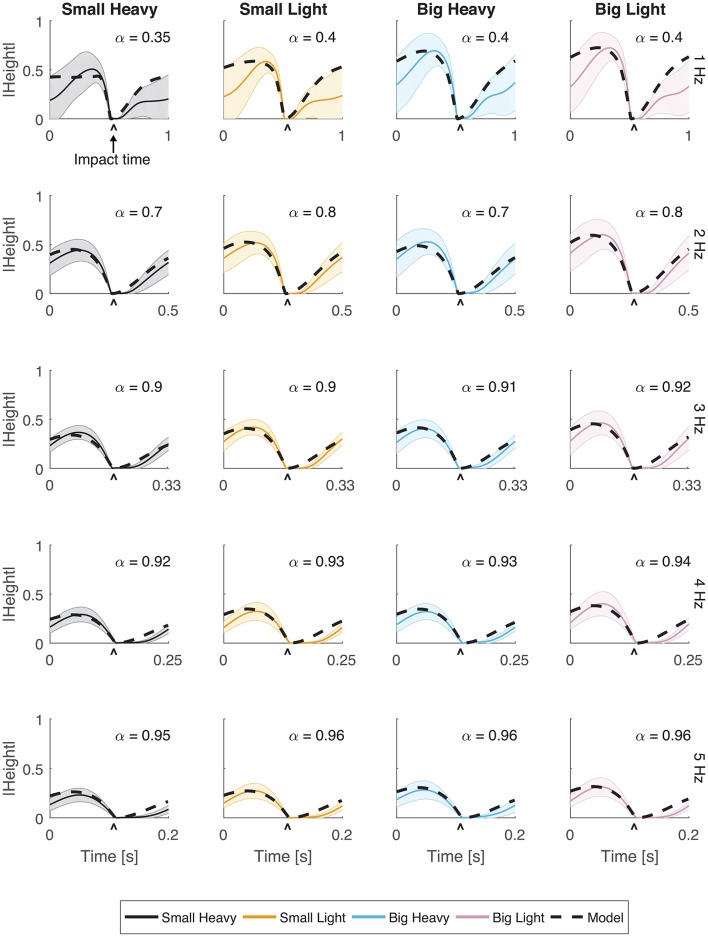
Vertical movement for all hammers and frequencies. The normalized vertical position of the hammer head was plotted with respect to time. Solid lines indicate the average trajectory while shading represents standard error. The black dashed line indicates the optimal behavior of the model using an estimated α parameter for frequencies 1, 2, 3, 4, and 5 Hz. The root mean squared error (RMSE) of the model for each case has a root mean squared error of *RMSE* < 0.1.

**Figure 3:**
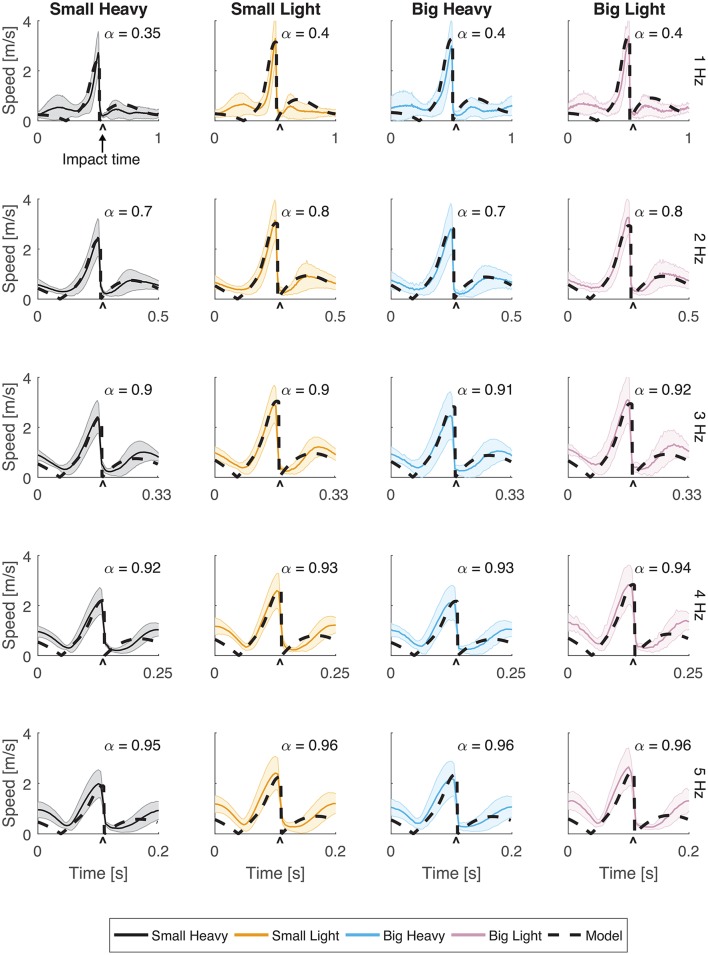
Speeds for all hammers and frequencies. The speed (magnitude of the velocity vector) of the hammer head was plotted with respect to time. Solid lines indicate the average speeds while shading represents standard deviation. The black dashed line indicates the optimal behavior of the model using an estimated α parameter for frequencies 1, 2, 3, 4, and 5 Hz. The root mean squared error (RMSE) of the model for each case has a root mean squared error of *RMSE* < 0.1 m/s.

Subjects were adept at matching hammering frequency with most of those dictated by the metronome. The hammering frequencies achieved by the subjects for metronome frequencies 1, 2, 3, 4, and 5 Hz were 0.99 ± 0.01, 2.02 ± 0.01, 3.01 ± 0.03, 4.01 ± 0.01, and 4.71 ± 0.04 Hz respectively (mean ± standard error). Hammering frequencies of 5 Hz were too fast for our subjects to reliably match. A hammering frequency of 1 Hz was uncomfortably slow for most subjects. To compensate, many subjects developed a strategy of pausing after each impact before initating an up-and-down hammering motion at a more comfortable frequency (Figures 2, 3).

Vertical trajectories (Figure 2) and speeds (Figure 3) exhibited by the subjects in hammering showed very few differences between the different hammers. However, decreasing the hammering frequency increased the variability in these movements. Rather than the single bell-shaped speed profile characteristic of reaching movements, subjects showed a bell-shaped speed profile for raising the hammer and another truncated bell-shaped speed profile for the descending motion (Figure 3).

**Figure 4:**
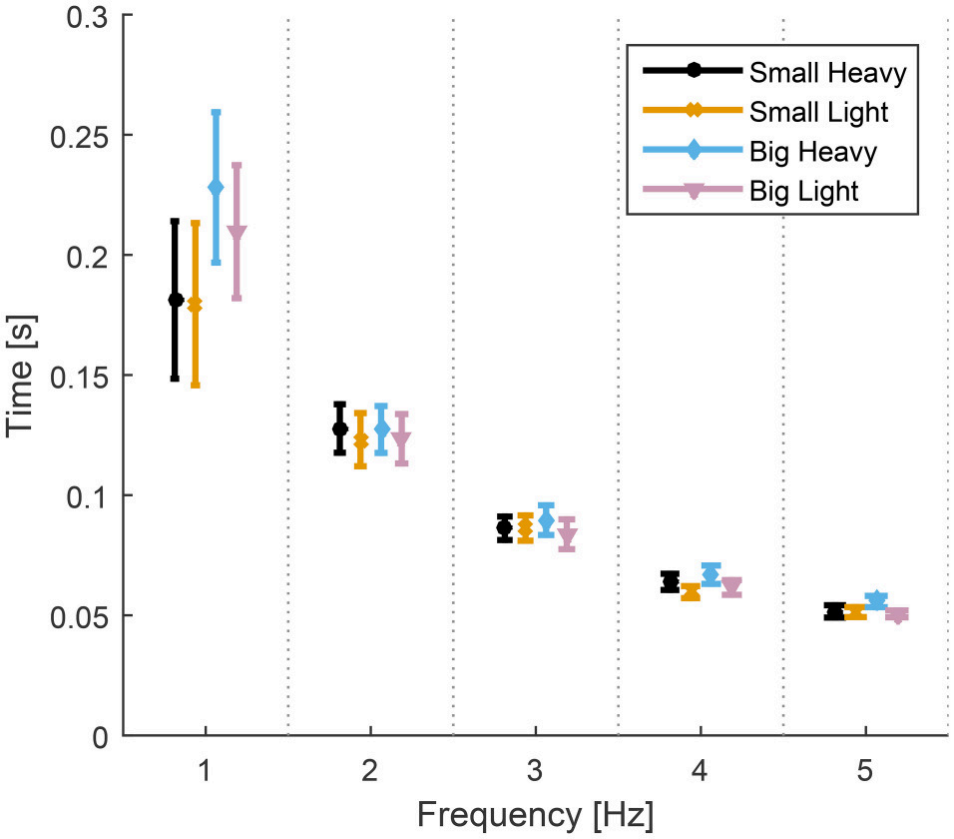
Means and standard errors (SEM) of time to impact for all hammers and frequencies. The time from maximal height to impact was statistically the same for all hammers at each hammering frequency despite some statistically different maximal heights (Figure 5). The time to impact decreases and becomes less variable as the hammering frequency increases.

Analysis of variance showed significant effects of both hammers [*F*_(1.61, 14.5)_ = 4.95, *p* = 0.03] and frequencies [*F*_(1.14, 20.25)_ = 22.35, *p* < 0.01] on the time to impact from maximum height. There was no significant interaction [*F*_(1.73, 16.05)_ = 2.56, *p* = 0.11] between the effects of hammers and frequencies on the time to impact from maximum height. The diagram in Figure 4 shows the means and standard errors (SEM) of time to impact for all hammers and frequencies.

**Figure 5:**
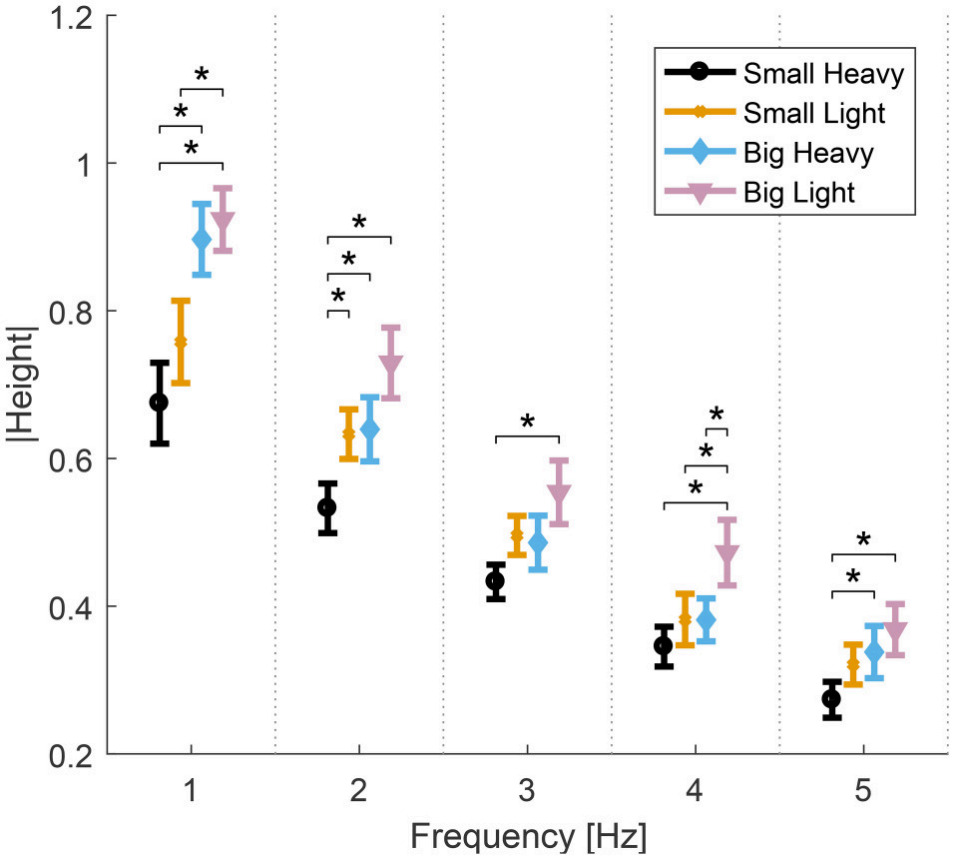
Means and standard errors (SEM) of the normalized maximal height of all hammers and frequencies. The normalized vertical height of the hammer head decreases as hammering frequency increases. Big Light and Small Heavy maximal heights were statistically different from any of the others (^*^p < 0.05).

Analysis of variance showed significant effects of hammers and frequencies on the normalized maximal heights. Significant effects of both hammers [*F*_(2.91, 26.2)_ = 22.8, *p* < 0.01], frequencies [*F*_(1.77, 15.99)_ = 53.09, *p* < 0.01] and interaction between hammers and frequencies [*F*_(4.14, 37.26)_ = 2.71, *p* = 0.04] were observed. Further analysis of the effects of hammers on normalized maximal heights showed significant effects of hammers [*F*_(3, 27)_ = 6.46−11.08, *p* < 0.01] in all frequencies. *Post-hoc t*-tests showed that Big Light and Small Heavy maximal heights were statistically different from any of the others [*t*_(9)_ = 2.97−5.75, *p* < 0.01]. The diagram in Figure 5 shows the means and standard errors (SEM) of normalized maximal heights for all hammers and frequencies.

**Figure 6:**
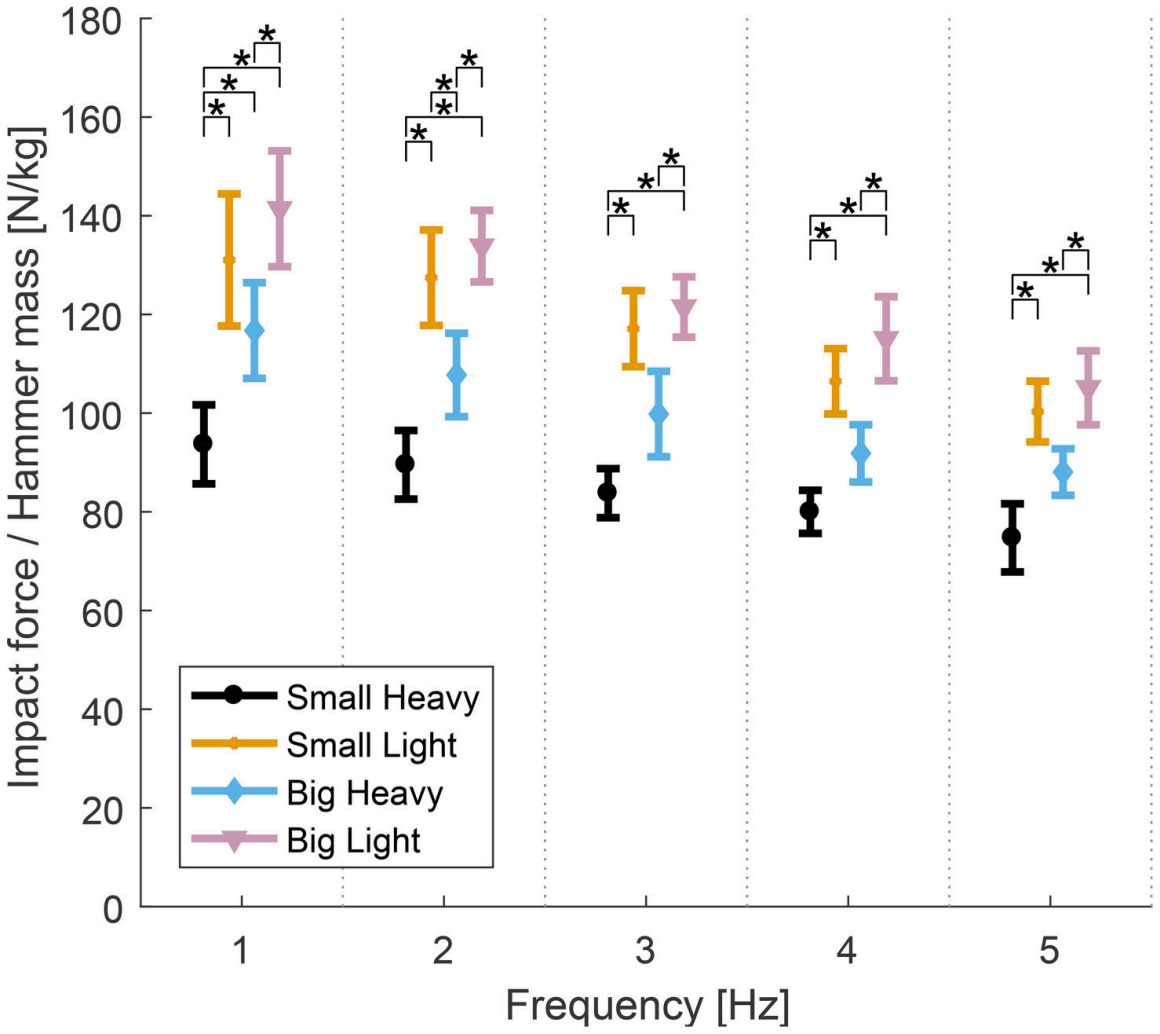
Means and standard errors (SEM) of the normalized maximal impact forces for all hammers and frequencies. Impact forces normalized by hammer mass varied between hammers. The heavy hammers generally had lower impact forces per unit mass than the lighter hammers across hammering frequencies. The Small Heavy hammer had the lowest normalized impact forces across conditions. The Big Light hammer produced the highest normalized impact forces, though the Big Heavy hammer produced the largest absolute impact forces. The Small Light and Big Heavy hammers were similar with statistical differences only found at 2 Hz (^*^p < 0.05).

Similarly, analysis of variance showed significant effects of hammers and frequencies on the impact forces normalized by hammer mass. Significant effects of both hammers [*F*_(2.06, 23.42)_ = 32.07, *p* < 0.01], frequencies [*F*_(1.34, 12.07)_ = 6.84], but no significant effect of interaction between hammers and frequencies [*F*_(4.39, 39.55)_ = 0.69, *p* = 0.63] were observed. Further analysis of the effects of hammers on the impact forces normalized by hammer mass showed significant effects of hammers [*F*_(3, 27)_ = 11.8−17.97, *p* < 0.01] in all frequencies. *Post-hoc t*-tests showed that Small Heavy was statistically different than Small Light [*t*_(9)_ = 4.71−5.65, *p* < 0.01] and Big Heavy [*t*_(9)_ = 5.4−6.61, *p* < 0.01], and Big Heavy was statistically different than Big Light [*t*_(9)_ = 3.04−3.91, *p* < 0.01] for all frequencies. The diagram in Figure 6 shows the means and standard errors (SEM) of impact forces normalized by hammer mass for all hammers and frequencies. The number of impacts needed to totally drive in the nail under each condition–a function of impact velocity–are reported in the appendix (Table A2).

### Modeling results

**Figure 7:**
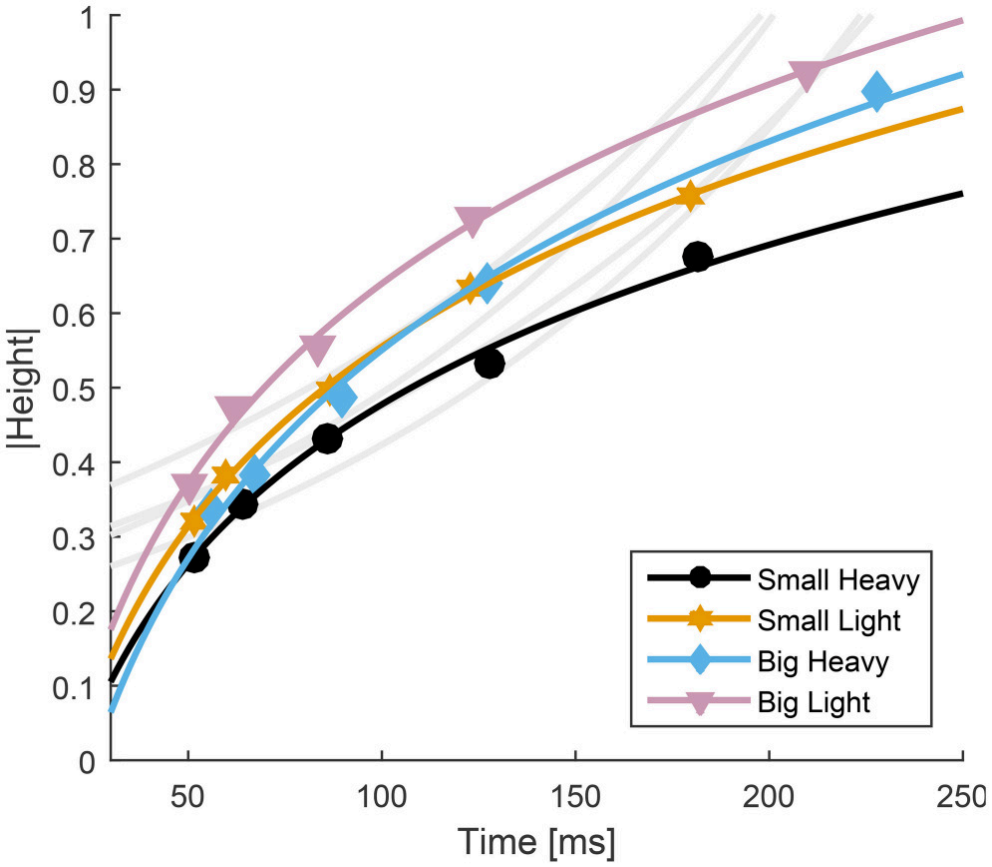
Relationship between time to impact and maximal height for all four hammers. The average normalized height was plotted with respect to average time to impact for each hammer. Fitts' Law was fit to the experimental data and overlayed on the experimental data (gray curves, *R*^2^ ≥ 0.88, *RMSE* ≤ 0.24). Because Fitts' Law appears to have opposite curvature to the experimental data, a modified model was developed (Equation 4) and overlayed on the experimental results (colored traces, *R*^2^ ≥ 0.99, *RMSE* ≤ 0.02).

Fitts' Law accurately predicted the movement time from the maximum height to impact (*R*^2^ ≥ 0.88). However, despite the high value of *R*^2^, the accepted formulation for Fitts' Law does not appear to follow the contours of the experimental data (Figure 7, light gray traces). Therefore, we propose a slightly altered model that reverses the relationship between movement time and distance to move and was able to improve upon Fitts' predictions (*R*^2^ ≥ 0.99),

(4) $$\begin{array}{r} D=W/2\;\left[a+b\cdot log_{2}\left(T_{f}\right)\right], \end{array}$$

where *D* is the maximal height of the hammer, *T*_*f*_ is the time from the maximal height to impact in milliseconds, *W* is the minimum width of the hammer face (our hammers were square, so both face length and width were the same), and *a* and *b* are parameters fit to the data using a least squares difference regression (Table 2).

**Table 2:** Parameter estimations for Equation (4) and the original Fitts' Law Equation (1).

**New model**	***a***	***b***	**SSE**	**RMSE**	***R*^2^**
Small heavy	−135	30.6	0.0005	0.015	0.99
Small light	−150	34.5	0.0001	0.007	0.99
Big heavy	−119	25.4	0.0012	0.020	0.99
Big light	−103	24.4	0.0008	0.016	0.99
**Fitts' model**	***a***	***b***	**SSE**	**RMSE**	*R*^2^
Small heavy	223	100	776	16	0.93
Small light	201	99	871	17	0.92
Big heavy	226	118	1,350	21	0.93
Big light	197	117	1,841	24	0.88

**Figure 8:**
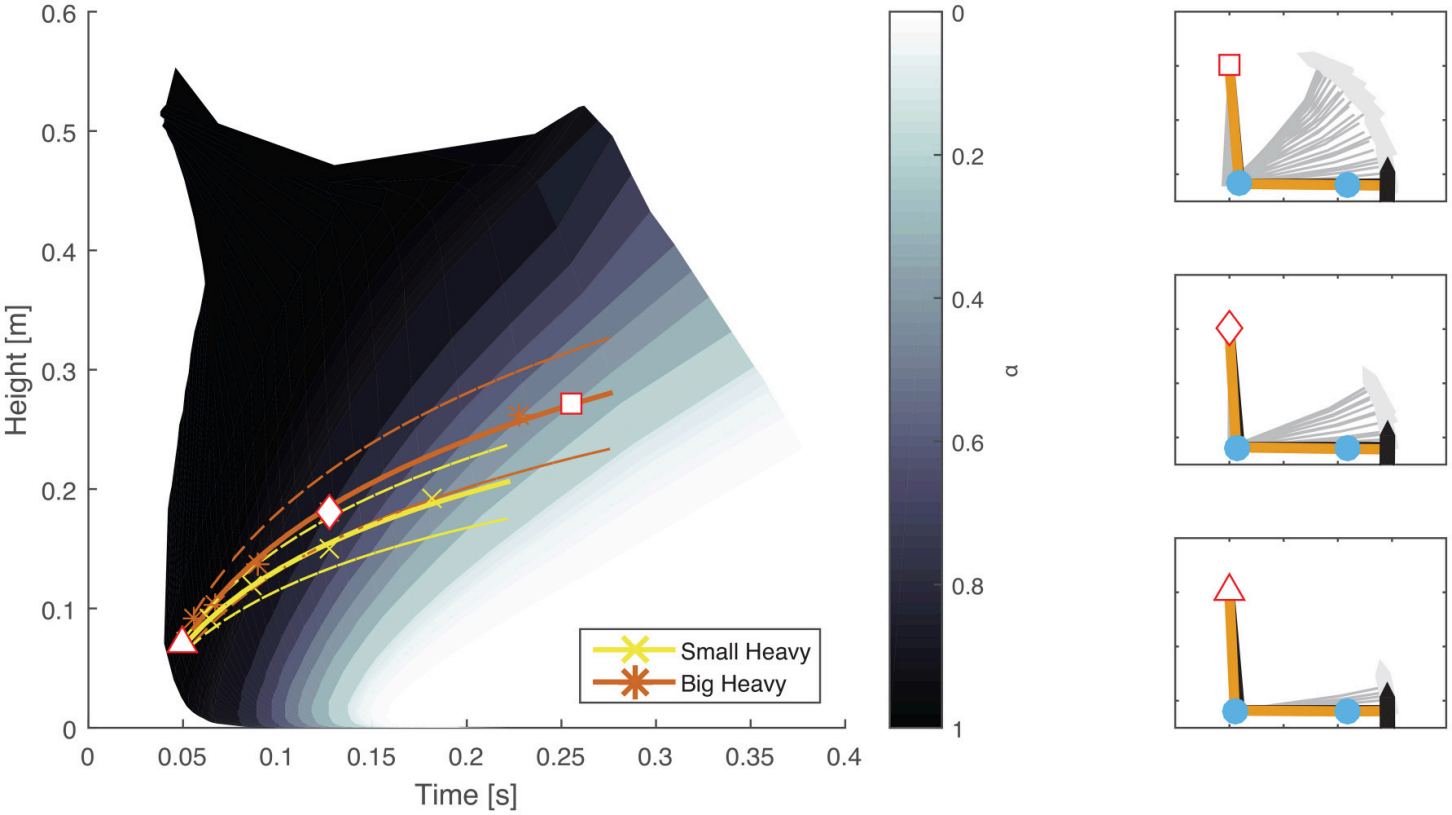
Comparison between experimental results and constant contours of α. A map showing the maximal height attained and time to impact generated using constant values of α (Equation 3) was overlayed on experimental results from two different hammers. Solid colored lines (yellow and orange) indicate mean experimental results while dashed lines indicate the standard error. A direct comparison shows that subjects emphasize effort conservation (low values of α) at low hammering frequencies (greater time between impacts) and energy transfer to the nail (high values of α) at high hammering frequencies (less time between impacts) rather than a constant relationship for all hammering speeds. The plots on the right hand side show examples of arm trajectories using different values of α. The specific values used are marked on the left hand plot by a white square, rhombus, and triangle for the top, middle, and bottom plots, respectively.

The optimal feedforward model was able to accurately reproduce the motions of the arm during hammering (Figures 2, 3, dashed lines, *RMSE* ≤ 0.1) using the cost function given by Equation (3). This model allows for the generation of optimal hammering trajectories by selecting just one parameter, α. This model also shows that subjects use roughly the same value of α for each hammer, despite the different properties of the different hammers (Figures 2, 3, α values in each row are very similar). The superpositioning of experimental data with computed contours of constant α values (Figure 8) showed that in practice subjects do not use a constant value of α for all hammering frequencies, but rather emphasize lower effort at slower hammering frequencies and energy transfer to the nail at faster hammering frequencies.

The Big Heavy hammer was the most preferred hammer followed by the Big Light, Small Heavy, and Small Light hammers in that order based on subject ratings (Table 3).

**Table 3:** Results of subjects' ranking of the hammers, e.g., S-H, Small Heavy; S-L, Small Light; B-H, Big Heavy; and B-L, Big Light.

**Ranking vs. Hammer**	**S-H**	**S-L**	**B-H**	**B-L**
Best (10)	0	0	9	1
7	3	1	1	5
3	3	4	0	3
Worst (1)	4	5	0	1
Score	3.4	2.4	9.7	5.5

## Discussion

The goal of this study was to examine the mechanics of a human upper extremity impact task and determine whether existing models of upper limb movement can explain the data. We found that subjects plan optimal trajectories that are a tradeoff between maximum impact velocity and minimal effort reminiscent of Fitts' Law and that are robust to different hammer conditions. However, we found that an altered version of Fitts' Law was able to better match the data than the typical formulation. We also found that end-effector speeds follow a “bell curve and a half” trajectory in hammering in which the hammer head moves upwards with a bell-shaped speed profile and then downwards with a bell-shaped profile before being truncated before the zenith of the curve (Figure 3).

Our analyses showed that Fitts' Law can be applied to human hammering (*R*^2^ ≥ 0.88). However, the large *R*^2^ values belie an apparent discrepancy between the curves generated using Fitts' Law and the experimental results (Figure 7, gray lines). Therefore, we identified a relationship between movement time and target distance that better reproduces the experimental data (Equation 4, *R*^2^ ≥ 0.99). In most Fitts' Law experiments, subjects are prescribed a reaching distance and are asked to move as fast as possible (Fitts, 1954). However, in our experiments, we constrain permitted movement time using the metronome and subjects were allowed to select the distance to reach. This difference may account for the relative effectiveness of our inverted formulation of Fitts' Law. However, other previous studies have reported violations of Fitts' Law (Adam et al., 2006; Glazebrook et al., 2015). Glazebrook et al. (2015) determined that these Fitts' Law violations are the result of pre-planning of movements. This explanation is also certainly plausible in the context of a cyclical task such as hammering. Finally, several studies have noted that Fitts' Law does not hold for movements in which subjects were not asked to move as quickly and as accurately as possible (Young et al., 2009). We do not explicitly instruct our subjects to move as quickly and accurately as possible. Instead, we instructed them to accomplish a task that is directly dependent on the speed of the movement and allow them to balance that movement speed with their motor capability, which we believe to be an approximation of the instructions to move as quickly and as accurately as possible. In hammering frequencies above 1 Hz, the computed values of α indicate that subjects weight movement speed very highly (Figures 2, 3), and thus likely approach a fast-as-possible movement for which Fitts' Law is presumed to be valid.

Our feedforward optimal hammering simulation was able to reproduce many of the features of human hammering (Figures 2, 3). Our simulations also allow us to show that humans prefer to emphasize energy transfer to the nail (larger values of α) when task constraints are high (high hammering frequencies) and minimal efforts (smaller values of α) when task constraints are low (low hammering frequencies; Figure 8). Our cost function was formulated to minimize the sum squared actuator effort, which serves to keep commanded joint torques small. These small actuation signals prevent excessive energy expenditure during the task (Crowninshield and Brand, 1981; Missenard and Fernandez, 2011), but this quadratic formulation might also serve to keep disturbances from motor noise whose effects are multiplicative with actuator effort small (Harris and Wolpert, 1998; Todorov and Li, 2005; Franklin and Wolpert, 2011). In this context, the adaptive prioritization that we observed (changing values of α) might be due to fewer task constraints permitting higher peak heights to be attained at slow hammering frequencies, thus increasing the potential for errors to accrue and increasing the relative importance of accuracy. While the exact cost function used by the nervous system cannot be known exactly, the current formulation reproduces many of the features observed in the experimental results including maximum heights attained, the general trajectories followed, and the robustness to different hammers (similar values of α for different hammers at the same hammering frequencies). However, this model failed to capture the latency after impact before initiating the upward movement of the hammer. This discrepancy may be due to compliance in the musculoskeletal system (e.g., series-elastic muscle-tendon units, Hill, 1938; Fung, 2013) that was not captured by our model.

Despite different hammer dynamics (Table 1), hammering kinematics were fairly uniform across many different cases with few statistical differences found between the different hammers in the time from maximal height to impact, maximal hammer height, and impact velocity. Previous studies have suggested that the redundancy of the human musculoskeletal system (Bernstein, 1967) may contribute to considerable robustness to slight changes in dynamics (Martelli et al., 2015; Simpson et al., 2015) or to dysfunction (Arnold et al., 2005; Hicks et al., 2008; Correa et al., 2012; Steele et al., 2012). While these studies rely on highly redundant lower body musculoskeletal models, other studies examining less redundant body parts have shown limited ability to compensate for dysfunction (Valero-Cuevas and Hentz, 2002; Kutch and Valero-Cuevas, 2011). However, detailed models of the upper extremity indicate muscular redundancy on the same level as detailed models of the lower body (Table 4) suggesting that similar robustness to perturbations might be expected. The human nervous system may also select control strategies that are purposefully robust (Mitrovic et al., 2010; Franklin and Wolpert, 2011), but our formulation does not include any such criteria, suggesting that consistent movement patterns across conditions might be due to embodied intelligence (e.g., redundant actuators and compliance).

**Table 4:** Musculoskeletal models used in examinations of robustness.

**Body part**	**Degrees of freedom**	**Number of muscles**	**References**
Upper extremity	15	50	Holzbaur et al., 2005
Lower body	23	54+	Delp et al., 1990, 2007
Index finger	4	7	Kutch and Valero-Cuevas, 2011
Simple leg	3	14	Kutch and Valero-Cuevas, 2011

Stiffness, or impedance, is a crucial parameter modulated by humans to stably interact with their environment (Burdet et al., 2001; Franklin and Wolpert, 2011). Impedance is difficult to record experimentally, but previous studies have attempted to estimate joint stiffnesses based on muscle properties (Hu et al., 2011), through simulation (Thelen et al., 2003), or by experimentally recording endpoint stiffnesses (Burdet et al., 2000, 2001). Because of practical limitations, measurements of muscle activity or impedance were not included in this study, but likely play an important role in impact tasks and should be considered in future works.

Despite the difficulty of controlling a highly nonlinear plant using noisy control signals and noisy sensors with variable delays in an uncertain environment, biological movement appears to be highly robust. However, robustness has not been well addressed in robot learning (Schaal and Atkeson, 2010; Nguyen-Tuong and Peters, 2011) primarily because it is difficult to design controllers that are robust to the model structure or parameter errors. One possible solution is to use control policies with optimization criteria based on biological models. For example, the tradeoff between maximizing task performance and accuracy could potentially serve as an optimization criteria for robot hammering.

In this paper, we have extracted the mechanics involved in a targeted upper extremity impact task and demonstrated that the human motor control strategies involved are robust to many different conditions including hammer mass, hammer face area, and timing constraints. We have shown that while many traditional models of human reaching hold for this novel task (bell-shaped speed profiles and Fitts' Law), an altered version of Fitts' Law can better match experimental results. We have also demonstrated that optimality principles previously demonstrated for reaching movements can be generalized to targeted impact tasks and thus lay a framework that can be used for the planning of targeted impact tasks in robots.

## Author contributions

TP, CS, AU, and AI contributed to the design, execution and drafting of this work, and approved the final manuscript. Experimental data was collected and analyzed by TP and CS.

### Conflict of interest statement

The authors declare that the research was conducted in the absence of any commercial or financial relationships that could be construed as a potential conflict of interest.

## Appendix

### Model parameters

In this paper, we have modeled the human arm holding a hammer as a torque driven 3 degree of freedom (DOF) robot operating in the sagittal plane. Each DOF is driven by an independently controlled torque generator capable of producing both positive and negative torques. The robot parameters (link lengths, center of mass locations, etc.) were computed based on data from Winter (2009) using the mean height (176.9 cm) and weight (77 kg) of subjects that participated in this study. The hammers were simulated by adding the relevant mass to the end effector. Parameters of the hammers are in Table 1. The robot parameters are given in Table A1.

**Table A1:** Parametes of the dynamical model.

**Part**	**Upper Arm**	**Lower Arm**	**Hand + Hammer**
Link No.	1	2	3
Length	0.2794	0.2667	0.0855
Mass	2.0751	1.2225	0.4810
Center of mass location	0.1613	0.1220	0.0676
Inertia	0.0131	0.0067	0.0010

*Note that all units are SI (m, kg). Note also that the center of mass location is relative to the proximal end of the relevant link*.

### Hammer hits

The table reports how many cycles were necessary to totally drive in the nail with respect to the hammer and frequency.

**Table A2:** Number of impacts required to drive nail by hammer and frequency (mean ± standard error).

		**Hammer**			
		**S-H**	**S-L**	**B-H**	**B-L**
Frequency	1	12 ± 5.114	9.9 ± 1.215	7.1 ± 1.663	8.5 ± 1.258
[Hz]	2	16 ± 3.303	12.2 ± 1.679	9.7 ± 2.285	13.4 ± 2.817
	3	19.7 ± 4.6	14.4 ± 2.579	10.6 ± 1.655	14.4 ± 1.968
	4	22 ± 3.48	19.5 ± 2.693	12.9 ± 2.089	14.1 ± 2.677
	5	29.2 ± 6.2	23.8 ± 3.62	14.2 ± 2.732	20.2 ± 3.511
